# 4-[3-(4-Nitro­phen­oxy)prop­oxy]aniline

**DOI:** 10.1107/S1600536808032406

**Published:** 2008-10-15

**Authors:** Li-Mei Zheng, Xian Wei, Xiao-Rong Peng, Jin-Ping Zeng, Yun-Qian Zhang

**Affiliations:** a School of Chemistry and Chemical Engineering, Henan University of Technology, Zhengzhou 450001, People’s Republic of China; b Key Laboratory of Macrocyclic and Supramolecular Chemistry of Guizhou Province, Guizhou University, Guiyang 550025, People’s Republic of China

## Abstract

The mol­ecules of the title compound, C_15_H_16_N_2_O_4_, are linked *via* N—H⋯O hydrogen bonds, forming undulating one-dimensional chains. Adjacent chains are linked by weak C—H⋯π inter­actions, forming a three-dimensional network.

## Related literature

For general background, see: Day & Arnold (2000 ▶); Day *et al.* (2002 ▶); Freeman *et al.* (1981 ▶); Kim *et al.* (2000 ▶).
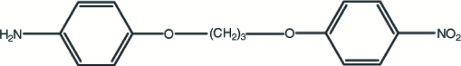


## Experimental

### 

#### Crystal data

C_15_H_16_N_2_O_4_
*M* *_r_* = 288.30
Orthorhombic, 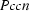

*a* = 10.808 (8) Å
*b* = 34.79 (3) Å
*c* = 7.596 (6) Å
*V* = 2857 (4) Å^3^
*Z* = 8
Mo *K*α radiation
μ = 0.10 mm^−1^
*T* = 298 (2) K
0.23 × 0.19 × 0.16 mm

#### Data collection

Bruker APEXII CCD area-detector diffractometer
Absorption correction: multi-scan (*SADABS*; Bruker, 2005 ▶) *T*_min_ = 0.978, *T*_max_ = 0.984
17736 measured reflections
2509 independent reflections
1554 reflections with *I* > 2σ(*I*)
*R*_int_ = 0.065

#### Refinement

*R*[*F* ^2^ > 2σ(*F* ^2^)] = 0.046
*wR*(*F* ^2^) = 0.126
*S* = 1.05
2509 reflections
190 parameters
H-atom parameters constrained
Δρ_max_ = 0.24 e Å^−3^
Δρ_min_ = −0.16 e Å^−3^

### 

Data collection: *APEX2* (Bruker, 2005 ▶); cell refinement: *SAINT* (Bruker, 2005 ▶); data reduction: *SAINT*; program(s) used to solve structure: *SHELXS97* (Sheldrick, 2008 ▶); program(s) used to refine structure: *SHELXL97* (Sheldrick, 2008 ▶); molecular graphics: *ORTEP-3 for Windows* (Farrugia, 1997 ▶); software used to prepare material for publication: *WinGX* (Farrugia, 1999 ▶).

## Supplementary Material

Crystal structure: contains datablocks global, I. DOI: 10.1107/S1600536808032406/at2638sup1.cif

Structure factors: contains datablocks I. DOI: 10.1107/S1600536808032406/at2638Isup2.hkl

Additional supplementary materials:  crystallographic information; 3D view; checkCIF report

## Figures and Tables

**Table 1:** Hydrogen-bond geometry (Å, °)

*D*—H⋯*A*	*D*—H	H⋯*A*	*D*⋯*A*	*D*—H⋯*A*
N2—H2*B*⋯O1^i^	0.86	2.29	3.123 (3)	164
C3—H3⋯*Cg*1^ii^	0.93	3.07	3.513 (4)	111
C7—H7*B*⋯*Cg*2^iii^	0.97	2.71	3.567 (4)	148
C13—H13⋯*Cg*2^iv^	0.93	3.01	3.757 (4)	139

Symmetry codes: (i) 
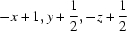
; (ii) 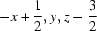; (iii) 
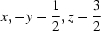
; (iv) 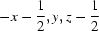. *Cg*1 and *Cg*2 are the centroids of the C1–C6 and C10–C15 phenyl rings, respectively

**Fig. 1.**
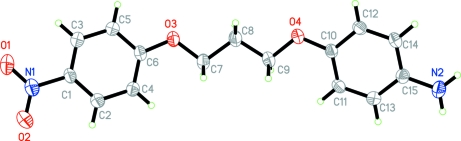
The molecular structure of (I), showing the atom-labelling scheme. Displacement ellipsoids are drawn at the 30% probability level.

**Fig. 2.**
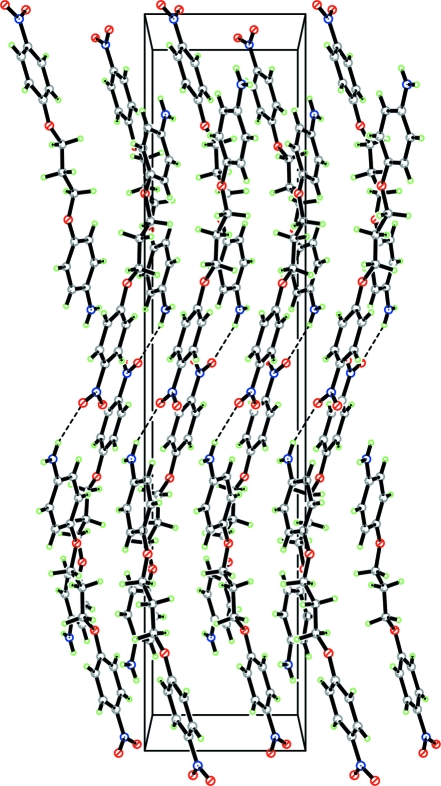
Packing diagram of (I). Hydrogen bonds are shown as dashed lines.

## supplementary crystallographic information

### Comment

As part of our ongoing investigation on bibenzene compound, we present the crystal structure of the title compound (I) containing multiple functional groups that can develop strong interactions with cucurbit[*n*]urils (CB[*n*]) (Freeman *et al.*, 1981; Day & Arnold, 2000; Day *et al.*, 2002; Kim *et al.*, 2000)

The crystal structure of (I) is shown in Fig.1. Two phenyl rings were linked by ethereal chain forming a non-coplanar structure and the dihedral angle between two phenyl ring is 26.13 (9) Å. Molecules are linked *via* N2—H2B···O1 hydrogen bonds forming a undulant one-dimensional chains (Fig. 2) and adjacent chains are linked by C—H···π interaction forming a three-dimensional framework (Table 1, *Cg*1 and *Cg*2 are centroids of the phenyl ring (C1—C6) and (C10—C15), respectively).

### Experimental

*P*-toluenesulfonyl chloride (7.62 g, 40 mmol) was added slowly, whilst stirring, to a pyridine solution (50 ml) containing 1,3-propanediol (1.52 g, 20 mmol). The mixture was stirred for about 4 h in the range of 268 K - 278 K. Water (40 ml) was added to the resulting solution, the precipitate was collected by filtration, the solid product was crystallized using ethanol. The solid product (6.85 g, 20 mmol) dissolved in DMF (100 ml) containing K_2_CO_3_ (2 g), *p*-nitrophenol (0.54 g, 4 mmol) was added slowly, to the DMF(100 ml) solution, and the mixture was heated at 353 K for 24 h, and then the solvent was removed into water and filtered, the residue was washed with water, and 1,3-bis(-nitrylphenoxy)-propane was obtained. Hydrazine (30 g,80%) was added slowly to a stirred solution of ethanol (50 ml) containing 1,4-bis(-nitrylphenoxy)-propane (3.12 g, 10 mmol), FeCl_3_.6H_2_O (0.8 g) and active carbon (1.8 g) at 348 K for 5 h, and then the solvent was filtered, the solid product was crystallized using ethanol, Single crystals of (I) were obtained after a week.

### Refinement

All H atoms were placed in calculated positions and refined as riding, with C—H = 0.97 Å (methylene) and 0.93 Å (aromatic), N—H = 0.861 Å, and *U*_iso_(H) = 1.2*U*_eq_(C,*N*).

### Crystal data

**Table:**

C_15_H_16_N_2_O_4_	*F*(000) = 1216
*M**_r_* = 288.30	*D*_x_ = 1.341 Mg m^−^^3^
Orthorhombic, *P**c**c**n*	Mo *K*α radiation, λ = 0.71073 Å
Hall symbol: -P 2ab 2ac	Cell parameters from 2509 reflections
*a* = 10.808 (8) Å	θ = 2.0–25.0°
*b* = 34.79 (3) Å	µ = 0.10 mm^−^^1^
*c* = 7.596 (6) Å	*T* = 298 K
*V* = 2857 (4) Å^3^	Prism, brown
*Z* = 8	0.23 × 0.19 × 0.16 mm

### Data collection

**Table:**

Bruker APEXII CCD area-detector diffractometer	2509 independent reflections
Radiation source: fine-focus sealed tube	1554 reflections with *I* > 2σ(*I*)
graphite	*R*_int_ = 0.065
φ and ω scans	θ_max_ = 25.0°, θ_min_ = 2.0°
Absorption correction: multi-scan (SADABS; Bruker, 2005)	*h* = −12→12
*T*_min_ = 0.978, *T*_max_ = 0.984	*k* = −37→41
17736 measured reflections	*l* = −8→9

### Refinement

**Table:**

Refinement on *F*^2^	Primary atom site location: structure-invariant direct methods
Least-squares matrix: full	Secondary atom site location: difference Fourier map
*R*[*F*^2^ > 2σ(*F*^2^)] = 0.046	Hydrogen site location: inferred from neighbouring sites
*wR*(*F*^2^) = 0.126	H-atom parameters constrained
*S* = 1.05	*w* = 1/[σ^2^(*F*_o_^2^) + (0.0596*P*)^2^] where *P* = (*F*_o_^2^ + 2*F*_c_^2^)/3
2509 reflections	(Δ/σ)_max_ = 0.001
190 parameters	Δρ_max_ = 0.25 e Å^−^^3^
0 restraints	Δρ_min_ = −0.16 e Å^−^^3^

### Special details

**Table:**

Geometry. All e.s.d.'s (except the e.s.d. in the dihedral angle between two l.s. planes) are estimated using the full covariance matrix. The cell e.s.d.'s are taken into account individually in the estimation of e.s.d.'s in distances, angles and torsion angles; correlations between e.s.d.'s in cell parameters are only used when they are defined by crystal symmetry. An approximate (isotropic) treatment of cell e.s.d.'s is used for estimating e.s.d.'s involving l.s. planes.
Refinement. Refinement of *F*^2^ against ALL reflections. The weighted *R*-factor *wR* and goodness of fit *S* are based on *F*^2^, conventional *R*-factors *R* are based on *F*, with *F* set to zero for negative *F*^2^. The threshold expression of *F*^2^ > σ(*F*^2^) is used only for calculating *R*-factors(gt) *etc*. and is not relevant to the choice of reflections for refinement. *R*-factors based on *F*^2^ are statistically about twice as large as those based on *F*, and *R*- factors based on ALL data will be even larger.

### Fractional atomic coordinates and isotropic or equivalent isotropic displacement parameters (Å^2^)

**Table:**

	*x*	*y*	*z*	*U*_iso_*/*U*_eq_	
C1	0.3694 (2)	0.52612 (6)	0.2758 (3)	0.0531 (6)	
C2	0.4692 (2)	0.54418 (6)	0.1976 (3)	0.0587 (6)	
H2	0.5427	0.5308	0.1793	0.070*	
C3	0.2592 (2)	0.54512 (6)	0.3021 (3)	0.0575 (6)	
H3	0.1926	0.5326	0.3541	0.069*	
C4	0.4590 (2)	0.58237 (6)	0.1467 (3)	0.0528 (6)	
H4	0.5259	0.5949	0.0950	0.063*	
C5	0.2486 (2)	0.58278 (6)	0.2507 (3)	0.0556 (6)	
H5	0.1743	0.5957	0.2676	0.067*	
C6	0.3483 (2)	0.60180 (6)	0.1735 (3)	0.0468 (5)	
C7	0.4269 (2)	0.66196 (6)	0.0598 (3)	0.0540 (6)	
H7A	0.4487	0.6531	−0.0571	0.065*	
H7B	0.4994	0.6600	0.1346	0.065*	
C8	0.3816 (2)	0.70308 (6)	0.0527 (3)	0.0577 (6)	
H8A	0.3102	0.7046	−0.0244	0.069*	
H8B	0.3558	0.7110	0.1695	0.069*	
C9	0.4804 (2)	0.73000 (5)	−0.0131 (3)	0.0539 (6)	
H9A	0.5580	0.7249	0.0468	0.065*	
H9B	0.4929	0.7264	−0.1384	0.065*	
C10	0.5238 (2)	0.79809 (6)	−0.0124 (3)	0.0459 (5)	
C11	0.6378 (2)	0.79332 (6)	−0.0955 (3)	0.0507 (6)	
H11	0.6630	0.7690	−0.1311	0.061*	
C12	0.4878 (2)	0.83474 (6)	0.0389 (3)	0.0493 (6)	
H12	0.4117	0.8383	0.0935	0.059*	
C13	0.7136 (2)	0.82491 (6)	−0.1252 (3)	0.0534 (6)	
H13	0.7894	0.8214	−0.1808	0.064*	
C14	0.5645 (2)	0.86604 (6)	0.0094 (3)	0.0512 (6)	
H14	0.5393	0.8903	0.0455	0.061*	
C15	0.6788 (2)	0.86171 (6)	−0.0736 (3)	0.0487 (5)	
N1	0.3792 (2)	0.48636 (6)	0.3322 (3)	0.0763 (7)	
N2	0.75855 (17)	0.89324 (5)	−0.0993 (3)	0.0694 (6)	
H2A	0.8298	0.8898	−0.1474	0.083*	
H2B	0.7361	0.9159	−0.0669	0.083*	
O1	0.29183 (19)	0.47143 (5)	0.4107 (3)	0.1127 (8)	
O2	0.4737 (2)	0.46834 (5)	0.3007 (4)	0.1200 (9)	
O3	0.32733 (13)	0.63919 (4)	0.1305 (2)	0.0590 (4)	
O4	0.44110 (13)	0.76862 (4)	0.0214 (2)	0.0570 (4)	

### Atomic displacement parameters (Å^2^)

**Table:**

	*U*^11^	*U*^22^	*U*^33^	*U*^12^	*U*^13^	*U*^23^
C1	0.0549 (15)	0.0357 (14)	0.0687 (15)	0.0006 (11)	−0.0019 (12)	0.0067 (11)
C2	0.0491 (15)	0.0477 (15)	0.0793 (17)	0.0004 (12)	0.0011 (12)	0.0025 (12)
C3	0.0561 (16)	0.0499 (15)	0.0664 (15)	−0.0045 (12)	0.0055 (12)	0.0072 (12)
C4	0.0490 (14)	0.0455 (14)	0.0640 (15)	−0.0046 (11)	−0.0003 (11)	0.0053 (11)
C5	0.0505 (15)	0.0487 (15)	0.0677 (15)	0.0011 (12)	0.0070 (12)	0.0050 (12)
C6	0.0545 (14)	0.0361 (13)	0.0496 (13)	−0.0013 (11)	−0.0073 (11)	0.0021 (10)
C7	0.0594 (15)	0.0428 (14)	0.0598 (14)	−0.0065 (11)	−0.0049 (12)	0.0052 (10)
C8	0.0602 (15)	0.0445 (14)	0.0684 (15)	−0.0038 (11)	−0.0088 (12)	0.0069 (11)
C9	0.0666 (16)	0.0383 (13)	0.0566 (14)	0.0025 (11)	−0.0009 (11)	0.0040 (10)
C10	0.0493 (14)	0.0380 (13)	0.0504 (12)	0.0008 (11)	−0.0028 (10)	0.0031 (10)
C11	0.0524 (14)	0.0404 (13)	0.0594 (14)	0.0082 (11)	0.0000 (12)	−0.0023 (10)
C12	0.0513 (14)	0.0453 (14)	0.0514 (13)	0.0054 (11)	0.0031 (10)	−0.0005 (10)
C13	0.0493 (14)	0.0524 (15)	0.0584 (14)	0.0026 (11)	0.0019 (11)	0.0001 (11)
C14	0.0615 (15)	0.0391 (13)	0.0530 (14)	0.0025 (11)	−0.0022 (12)	−0.0065 (10)
C15	0.0500 (14)	0.0465 (14)	0.0495 (12)	−0.0027 (11)	−0.0038 (11)	0.0005 (10)
N1	0.0723 (17)	0.0485 (15)	0.1081 (18)	0.0008 (12)	0.0043 (14)	0.0138 (12)
N2	0.0672 (13)	0.0544 (13)	0.0867 (15)	−0.0160 (11)	0.0080 (11)	−0.0116 (11)
O1	0.0956 (16)	0.0661 (13)	0.176 (2)	−0.0062 (11)	0.0244 (15)	0.0501 (13)
O2	0.0921 (15)	0.0634 (14)	0.205 (3)	0.0244 (12)	0.0344 (16)	0.0389 (14)
O3	0.0557 (9)	0.0410 (9)	0.0805 (11)	−0.0021 (7)	−0.0004 (8)	0.0110 (8)
O4	0.0583 (10)	0.0381 (9)	0.0745 (11)	0.0006 (8)	0.0061 (8)	0.0040 (7)

### Geometric parameters (Å, °)

**Table:**

C1—C3	1.377 (3)	C9—O4	1.433 (2)
C1—C2	1.383 (3)	C9—H9A	0.9700
C1—N1	1.453 (3)	C9—H9B	0.9700
C2—C4	1.388 (3)	C10—O4	1.385 (2)
C2—H2	0.9300	C10—C12	1.389 (3)
C3—C5	1.372 (3)	C10—C11	1.393 (3)
C3—H3	0.9300	C11—C13	1.390 (3)
C4—C6	1.390 (3)	C11—H11	0.9300
C4—H4	0.9300	C12—C14	1.387 (3)
C5—C6	1.394 (3)	C12—H12	0.9300
C5—H5	0.9300	C13—C15	1.391 (3)
C6—O3	1.361 (3)	C13—H13	0.9300
C7—O3	1.440 (2)	C14—C15	1.394 (3)
C7—C8	1.513 (3)	C14—H14	0.9300
C7—H7A	0.9700	C15—N2	1.409 (3)
C7—H7B	0.9700	N1—O2	1.221 (2)
C8—C9	1.506 (3)	N1—O1	1.232 (3)
C8—H8A	0.9700	N2—H2A	0.8600
C8—H8B	0.9700	N2—H2B	0.8600
			
C3—C1—C2	121.3 (2)	O4—C9—H9A	110.1
C3—C1—N1	118.6 (2)	C8—C9—H9A	110.1
C2—C1—N1	120.1 (2)	O4—C9—H9B	110.1
C1—C2—C4	119.5 (2)	C8—C9—H9B	110.1
C1—C2—H2	120.2	H9A—C9—H9B	108.4
C4—C2—H2	120.2	O4—C10—C12	116.53 (19)
C5—C3—C1	119.3 (2)	O4—C10—C11	124.51 (19)
C5—C3—H3	120.4	C12—C10—C11	119.0 (2)
C1—C3—H3	120.4	C13—C11—C10	120.0 (2)
C2—C4—C6	119.6 (2)	C13—C11—H11	120.0
C2—C4—H4	120.2	C10—C11—H11	120.0
C6—C4—H4	120.2	C14—C12—C10	120.5 (2)
C3—C5—C6	120.6 (2)	C14—C12—H12	119.7
C3—C5—H5	119.7	C10—C12—H12	119.7
C6—C5—H5	119.7	C11—C13—C15	121.5 (2)
O3—C6—C4	124.99 (19)	C11—C13—H13	119.3
O3—C6—C5	115.26 (19)	C15—C13—H13	119.3
C4—C6—C5	119.8 (2)	C12—C14—C15	121.17 (19)
O3—C7—C8	106.95 (18)	C12—C14—H14	119.4
O3—C7—H7A	110.3	C15—C14—H14	119.4
C8—C7—H7A	110.3	C13—C15—C14	117.81 (19)
O3—C7—H7B	110.3	C13—C15—N2	120.8 (2)
C8—C7—H7B	110.3	C14—C15—N2	121.4 (2)
H7A—C7—H7B	108.6	O2—N1—O1	121.3 (2)
C9—C8—C7	111.73 (19)	O2—N1—C1	119.5 (2)
C9—C8—H8A	109.3	O1—N1—C1	119.2 (2)
C7—C8—H8A	109.3	C15—N2—H2A	120.0
C9—C8—H8B	109.3	C15—N2—H2B	120.0
C7—C8—H8B	109.3	H2A—N2—H2B	120.0
H8A—C8—H8B	107.9	C6—O3—C7	119.42 (17)
O4—C9—C8	108.19 (18)	C10—O4—C9	117.95 (17)
			
C3—C1—C2—C4	0.9 (3)	C10—C11—C13—C15	0.0 (3)
N1—C1—C2—C4	−179.1 (2)	C10—C12—C14—C15	0.6 (3)
C2—C1—C3—C5	−0.4 (4)	C11—C13—C15—C14	0.0 (3)
N1—C1—C3—C5	179.5 (2)	C11—C13—C15—N2	177.85 (19)
C1—C2—C4—C6	−0.6 (3)	C12—C14—C15—C13	−0.3 (3)
C1—C3—C5—C6	−0.3 (3)	C12—C14—C15—N2	−178.14 (19)
C2—C4—C6—O3	179.49 (19)	C3—C1—N1—O2	175.7 (2)
C2—C4—C6—C5	−0.1 (3)	C2—C1—N1—O2	−4.4 (4)
C3—C5—C6—O3	−179.1 (2)	C3—C1—N1—O1	−4.1 (4)
C3—C5—C6—C4	0.6 (3)	C2—C1—N1—O1	175.8 (2)
O3—C7—C8—C9	177.80 (17)	C4—C6—O3—C7	−3.4 (3)
C7—C8—C9—O4	−167.07 (17)	C5—C6—O3—C7	176.24 (18)
O4—C10—C11—C13	179.26 (19)	C8—C7—O3—C6	−171.25 (17)
C12—C10—C11—C13	0.3 (3)	C12—C10—O4—C9	−173.68 (17)
O4—C10—C12—C14	−179.64 (18)	C11—C10—O4—C9	7.3 (3)
C11—C10—C12—C14	−0.6 (3)	C8—C9—O4—C10	173.62 (17)

### Hydrogen-bond geometry (Å, °)

**Table:**

*D*—H···*A*	*D*—H	H···*A*	*D*···*A*	*D*—H···*A*
N2—H2B···O1^i^	0.86	2.29	3.123 (3)	164
C3—H3···Cg1^ii^	0.93	3.07	3.513 (4)	111
C7—H7B···Cg2^iii^	0.97	2.71	3.567 (4)	148
C13—H13···Cg2^iv^	0.93	3.01	3.757 (4)	139

Symmetry codes: (i) −*x*+1, *y*+1/2, −*z*+1/2; (ii) −*x*+1/2, *y*, *z*−3/2; (iii) *x*, −*y*−1/2, *z*−3/2; (iv) −*x*−1/2, *y*, *z*−1/2.
